# Targeting RFWD2 as an Effective Strategy to Inhibit Cellular Proliferation and Overcome Drug Resistance to Proteasome Inhibitor in Multiple Myeloma

**DOI:** 10.3389/fcell.2021.675939

**Published:** 2021-04-21

**Authors:** Mengjie Guo, Pinggang Ding, Zhen Zhu, Lu Fan, Yanyan Zhou, Shu Yang, Ye Yang, Chunyan Gu

**Affiliations:** ^1^School of Medicine & Holistic Integrative Medicine, Nanjing University of Chinese Medicine, Nanjing, China; ^2^Large Data Center, Nanjing Hospital of Chinese Medicine affiliated to Nanjing University of Chinese Medicine, Nanjing, China; ^3^College of Health and Rehabilitation & College of Acupuncture and Massage, Nanjing University of Chinese Medicine, Nanjing, China

**Keywords:** multiple myeloma, RFWD2, proliferation, drug resistance, P27, ubiquitination, RCHY1

## Abstract

The potential to overcome resistance to proteasome inhibitors is greatly related with ubiquitin-proteasome system during multiple myeloma (MM) treatment process. The constitutive photomorphogenic 1 (RFWD2), referred to an E3 ubiquitin ligase, has been identified as an oncogene in multiple cancers, yet important questions on the role of RFWD2 in MM biology and treatment remain unclear. Here we demonstrated that MM patients with elevated RFWD2 expression achieved adverse outcome and drug resistance by analyzing gene expression profiling. Moreover, we proved that RFWD2 participated in the process of cell cycle, cell growth and death in MM by mass spectrometry analysis. *In vitro* study indicated that inducible knockdown of RFWD2 hindered cellular growth and triggered apoptosis in MM cells. Mechanism study revealed that RFWD2 controlled MM cellular proliferation via regulating the degradation of P27 rather than P53. Further exploration unveiled that RFWD2 meditated P27 ubiquitination via interacting with RCHY1, which served as an E3 ubiquitin ligase of P27. Finally, *in vivo* study illustrated that blocking RFWD2 in BTZ-resistant MM cells overcame the drug resistance in a myeloma xenograft mouse model. Taken together, these findings provide compelling evidence for prompting that targeting RFWD2 may be an effective strategy to inhibit cellular proliferation and overcome drug resistance to proteasome inhibitor in MM.

## Introduction

The uncontrolled expansion of plasma cells has been pinpointed as the major feature of multiple myeloma (MM), which synthesize and excrete a substantial amount of paraproteins (Gandolfi et al., 2017). In order to avoid the accumulation of the proteins involved in tumor pathogenesis, the MM cells are largely reliant on proteasome complexes, especially on the 26S proteasome, which is responsible for degrading intracellular proteins through ubiquitination pathway (Gandolfi et al., 2017). Therefore, MM cells are more sensitive to proteasome inhibition. Proteasome inhibitors (PIs) have emerged as an effective therapy for the treatment of MM patients in the past two decades (Richardson et al., 2018), which trigger endoplasmic reticulum stress to induce MM cell apoptosis. The three classic PIs like bortezomib (BTZ), carfilzomib (CFZ) and ixazomib (IXZ), as well as the novel PIs under clinical investigation including marizomib and oprozomib, have been used in combination with other regimens, which have formed one of the backbones of treatment paradigm throughout the whole course of MM (Song et al., 2019). However, current therapy might result in unideal effects and the acquisition of drug resistance. Consequently, the prospect of overcoming drug resistance has made the ubiquitin (Ub) plus proteasome system (UPS) as a potential therapeutic target in MM.

An attractively therapeutic strategy for treating MM is focusing on non-proteasomal components within the UPS, such as the E3 ubiquitin ligases, determining the substrate selectivity for ubiquitination and degradation (Snoek et al., 2013). Current evidence demonstrates that overexpression or mutation of E3 ubiquitin ligases could drive tumor development (Huang et al., 2020). In our previous research, we identified an E3 ubiquitin ligase, known as the gene constitutive photomorphogenic 1 (RFWD2, also called COP1) (Gu et al., 2020). Multiple literature have reported that RFWD2 is engaged in tumorigenesis via meditating several biological processes like transcription, DNA repair, cell cycle arrest and apoptosis (Migliorini et al., 2011; Zou et al., 2017; Abbastabar et al., 2018). Since both tumor suppressor (like p53) and oncogene (like JUN) are among putative targets of RFWD2, the potential role of RFWD2 in a wide variety of cancers remains controversial (Song et al., 2020). Few reports showed a tumor suppressor role of RFWD2 in prostate cancer and gastric cancer (Vitari et al., 2011; Sawada et al., 2013). Conversely, RFWD2 was regarded as a tumor promoter in human hepatocellular carcinoma, breast cancer, ovarian adenocarcinoma and acute myeloid leukemia (Dornan et al., 2004a; Lee et al., 2010; Yoshida et al., 2013). One study by our group has demonstrated that inducible upregulation of RFWD2 is closely associated with myeloma cellular proliferation and contributes to PIs resistance (Gu et al., 2020). To complement the studies on RFWD2 overexpression with the inverse experiment, the action mode of depletion of endogenous RFWD2 in MM needs to be further explored.

The cyclin/CDK2 inhibitor P27 has been recognized as a vitally negative regulator of cell cycle, which disrupts the G1-to-S phase cell cycle transition (Yoon et al., 2019), functioning as a tumor suppressor. Aberrant activities of P27 cause abnormal alterations in cell cycle regulation and alleviate P27-suppressed target genes, which contribute to uncontrolled cell proliferation, thereby inducing tumors (Li et al., 2018). It has been well documented that the expression of P27 is mainly dominated by its rate of proteasome degradation, making E3 ubiquitin ligases as the key regulators involved in targeting P27 (Egozi et al., 2007; Rodriguez et al., 2020). RFWD2 serves as a negative regulator of P27 (Ko et al., 2019), leading to CSN6-mediated P27 degradation in HCT116 and HEK-293T cells (Choi et al., 2015a). Consistently, our previous work initially illustrated the interaction between RFWD2 and P27 (Gu et al., 2020). To intensively delineate the precise mechanisms associated with RFWD2-induced drug resistance in MM via targeting P27, we continued to investigate which E3 ubiquitin ligases involving P27 degradation interacted with RFWD2.

Heartened by the current studies on the biological aggressiveness of RFWD2 in various cancers, we herein continued with the previous findings in the impact of RFWD2 on MM progression and drug resistance, further proved that targeting RFWD2 could work as a potential treatment approach for MM.

## Materials and Methods

### Database Analysis

Message levels of RFWD2 in MM were determined using the gene expression profiling (GEP) cohorts, which were mined from the GEO database as previously described (Zhou et al., 2013). The outcome data were based on Total therapy 2 (TT2, GSE2658), TT3 (GSE2658), and the evaluation of proteasome inhibition for extending remission (APEX, GSE9782). The Dutch-Belgian Cooperative Trial Group for Hematology Oncology Group-65 (HOVON65) trials was collected from GSE19784.

### Antibodies and Reagents

Antibodies were purchased from Abcam (Cambridge, Cambs, United Kingdom) (RFWD2, catalog number ab56400; KPC2, catalog number ab177519) or ProteinTech Group (Chicago, IL, United States) (P27, catalog number25614-1-AP). Other antibodies were purchased from Cell Signaling Technology (Danvers, MA, United States). Rabbit IgG (a7016), mouse IgG (a7028) and doxycycline (DOX) were obtained from Beyotime Institute of Biotechnology (Shanghai, China). Bortezomib (BTZ) and other chemical reagents were obtained from Shanghai Aladdin Bio-Chem Technology (Shanghai, China).

### Cell Lines and Culture

Human MM cell lines, ARP1, H929, RPMI 8226, ANBL6, OCI-MY5, JJN3, XG1, U266 and MM1S were maintained in RPMI-1640 (Biological In-dustries, Kibbutz Beit Haemek, Israel), supplemented with 10% fetal bovine serum (Biological In-dustries, Kibbutz Beit Haemek, Israel), 100 U/mL penicillin and 100 μg/mL streptomycin (Sigma, St. Louis, MO). 293T cells were cultured in DMEM (Hyclone, Los Angeles, CA, United States). The BTZ-resistant MM cell lines, 8226/BTZ were produced by increasing BTZ concentration gradient in our institute. All cells were propagated *in vitro* under the condition of 37°C in a humidified atmosphere containing 5% CO_2_.

### Plasmids and Transfection

The plasmids including the human RFWD2 cDNA or shRNA cassettes were obtained from Generay Biotech (Shanghai, China). The RFWD2 cDNA was cloned into the lentiviral vector, CD513B-1. Under the control of a DOX-inducible gene promoter, RFWD2-targeted shRNA was cloned into the vector of pTRIPZ. Lenti-viruses containing cDNA or shRNA were created by co-transfection of the CD513B-1-RFWD2 vector or RFWD2 shRNA vector with packaging vectors (PLP1, PLP2, and PLP-VSVG) into 293T cells (attained 70-80% confluency) using Lipofectamine2000 Transfection Reagent. The virus supernatant was collected after 48 h and stored at −80°C, which were used for subsequent experiments. MM cells were transfected with the lentivirus and selected by puromycin treatment. Transduction efficiency was validated by Quantitative Real time-PCR assays (qPCR) or western blotting (WB).

### Myeloma Xenografts in NOD-SCID Mice

8226 WT, 8226/BTZ, 8226 RFWD2 KD and 8226/BTZ RFWD2 KD cells (5 × 10^6^) were injected subcutaneously into the left and right abdominal flanks of 6-8 weeks old NOD-SCID mice, respectively. On day 3 after injection, DOX (2 mg/mL) was employed on mice through drinking to induce the reduction of RFWD2. On day 7 mice were treated with intraperitoneal (IP) administrations of BTZ (1 mg/kg) twice weekly.

Tumor diameter was measured 2-3 times weekly by using calipers. Mice were sacrificed by IP injection of chloral hydrate and then tumor tissues were collected, weighed, photographed and stored frozen in case the tumor diameter reached 20 mm. All experimental procedures were performed in accordance with government-published recommendations for the Care and Use of laboratory animals and approved by the guidelines of Institutional Ethics Review Boards of Nanjing University of Chinese Medicine (Ethics Registration no. 201905A003) (Zhou et al., 2013).

### Cell Proliferation and Viability Assay

Cell viability was evaluated using Thiazolyl Blue Tetrazolium Bromide (MTT) assay, which was performed according to the manufacturer’s instructions (Beijing Solarbio Science & Technology) (Yuan et al., 2018). Cells were cultured in 96-well plates at a density of 1 × 10^4^ cells/well with repeats for 3 wells in each group. Absorbance was read at 570 nM using microplate reader (Thermo Fisher Scientific).

### Flow Cytometric Analysis of Cell Apoptosis

APC 5-Bromo-2′-Deoxyuridine (BrdU) Flow Kit (BD Pharmingen) was used to measure the stage of apoptosis and cell cycle by a FlowSight flow cytometer. Briefly, cells were resuspended with 195 μL staining buffer, and then added 5 μL (0.125 μg) of APC-BrdU antibody per well, and incubated at 4°C for 30 min in the dark. 488 nm excitation wavelength and 520 nm emission wavelength were termed as the working condition of FlowSight flow cytometer.

### WB and Co-immunoprecipitation (Co-IP)

Protein levels were determined by WB analysis under the procedure as previous described (Yang et al., 2018). Co-IP was performed according to the instructions of the Pierce Direct Magnetic IP/Co-IP kit as mentioned (Gu et al., 2016). As the RFWD2 cDNA used in the current study carrying the FLAG tag, FLAG antibody was used instead of RFWD2 antibody for IP. And the IgG antibody sharing the same host with the IP antibody was chosen as a negative control.

### *In vitro* Ubiquitylation Assay

MM cells were incubated with 20 μM MG132 (a proteasome inhibitor) for 12 h before collection, and lysed in IP lysis buffer. Afterward, the cell lysate was subjected to immunoprecipitation with an ubiquitin antibody, and immunoprecipitation was subsequently separated by SDS-PAGE and immunoblotted with a P27 antibody to detect the ubiquitination level of P27 (Wang et al., 2019).

### Mass Spectrometry (MS) Analysis

SDS-PAGE was used to separate proteins in ARP1 WT & OE cells, and gel bands at the expected size were excised and digested with sequencing-grade trypsin (Promega, United States). The MS was performed by Lianchuan Biotech (Hangzhou, China), which was conducted by using LC-MS technology (Q-Exactive, Thermo). The first process was to quantify the protein and then open the three-dimensional structure of the protein by reductive alkylation. After enzymolysis, the peptides were extracted, and MS was used to obtain the mass spectra of these peptides. Finally, the peptides were identified by the related software.

### Statistical Analysis

All data were expressed as means ± SD. The statistical analysis was carried out using GraphPad Prism 6.01 or SPSS 22.0 version. Two-tailed Student’s *t*-test (2 groups) and one-way analysis of variance (≥ 3 groups) were employed to determine the significant differences among experimental groups. The survival data were plotted using Kaplan-Meier curve and sketched by log-rank test. Hazard ratios were estimated using Cox’s proportional hazard model. Array CGH data analysis building on the Agilent 180,000-feature human CGH microarray was performed as described previously (Zhou et al., 2013). Significance was set at *P* < 0.05. *P* < 0.05 was labeled as^∗^, *P* < 0.01 as^∗∗^.

## Results

### Increased RFWD2 Expression Is Correlated With Poor Survival and Relapse in MM

**FIGURE 1:**
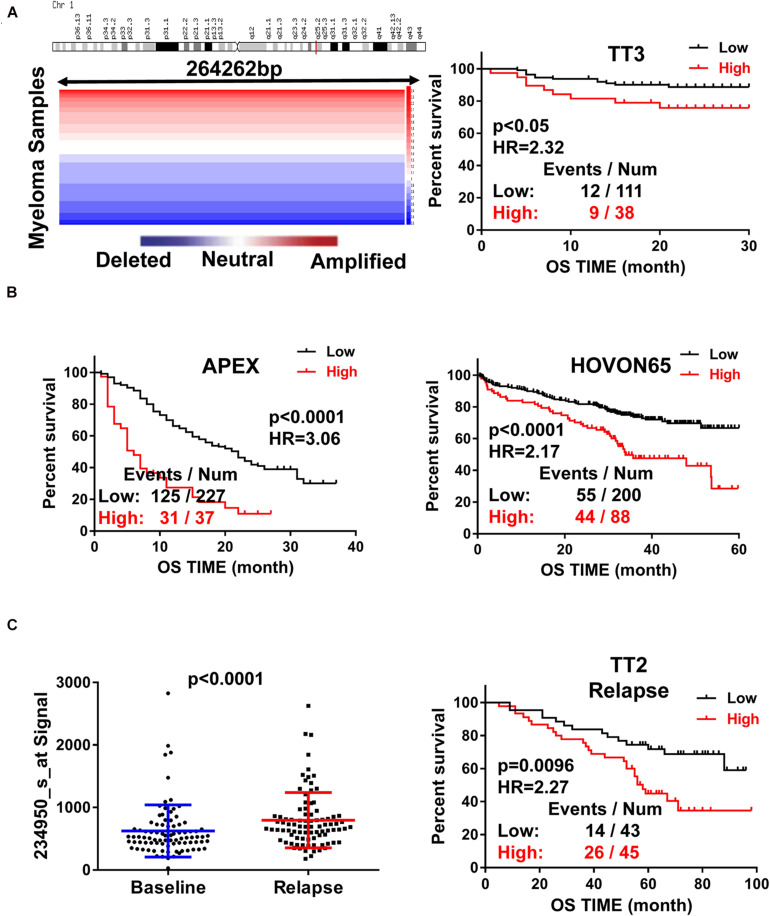
Increased RFWD2 expression is correlated with poor survival and relapse in MM. **(A)** Left: Array-based comparative genomic hybridization analysis illustrated RFWD2 copy number variation in 67 primary MM samples; Right: MM patients with high RFWD2 level were positively associated with poor overall survival (OS) in TT3cohort. **(B)** MM patients with elevated RFWD2 level exhibited positive correlation with poor overall survival (OS) in APEX and HOVON65 cohorts. **(C)** Left: RFWD2 expression in relapsed MM patients was significantly elevated compared with the corresponding newly diagnosed samples. Right: upregulation of RFWD2 was correlated with decreased OS in relapsed TT2 patients. The data were expressed as mean ± SD.

To assess the role of RFWD2 in MM, we analyzed the array-based comparative genomic hybridization (aCGH) data gained from 67 MM patients and found that RFWD2 locus was amplified in MM patient samples to a major extent (Figure 1A left). To determine the clinical significance of RFWD2 in MM, the prognosis of patients was best captured by analyzing GEP cohorts collected from the GEO database. As expected, Kaplan-Meier survival curves showed that MM patients with amplification of RFWD2 were significantly associated with poor overall survival (OS) in 3 independent MM cohorts (TT3, APEX and HOVON65) [Figures 1A,B (A-right)], which were in sync with the results of TT2 (a well-annotated, mature data set) and GMMG-HD4 cohort (Gu et al., 2020). Moreover, we found that elevated RFWD2 expression was impressively germane to clinical parameters, such as β2-microglobulin, hemoglobin concentration, and high-risk genetic parameters, such as chromosomal abnormalities (by G-banding) and g70high37 (*P* < 0.05; Table 1). It indicated that abnormal elevation of RFWD2 in MM leads to poor prognosis. Then we compared RFWD2 expression among 88 paired baseline/relapse samples. As illustrated in Figure 1C left, the RFWD2 expression in the relapse samples exhibited a dramatic upward trend compared with the corresponding newly diagnosed samples (*P* < 0.0001). Furthermore, overexpression of RFWD2 prognosticated inferior OS in the relapsed MM patients (*P* = 0.0096; Figure 1C right). These findings consolidate that RFWD2 acts as a valuable prognostic biomarker even in relapsed MM.

**TABLE 1:** The Correlation of RFWD2 Expression and Clinical Characteristics in TT2.

Characteristics	High RFWD2	Low RFWD2	

	(%, *n* = 186)	(%, *n* = 165)	*p* Value
Age at least 65 years	25.3	18.2	0.122
Female sex	42.5	44.2	0.747
White race	90.3	86.7	0.315
IgA isotype	28.4	23.0	0.271
CRP at least 4.0 mg/L	6.52	5.45	0.822
β2-Microglobulin at least 4.0 mg/L	42.5	25.4	0.001
Creatinine at least 2.0 mg/dL	14.0	8.64	0.129
Hemoglobin less than 10 g/dL	31.1	18.8	0.009
Albumin less than 3.5 g/dL	37.1	35.7	0.825
Chromosomal abnormalities (by G-banding)	40.3	29.7	0.044
MRI focal bone lesions, at least three	59.8	57.2	0.659
LDH at least 190 IU/L	37.5	30.3	0.175
Hyperdiploid	18.3	18.8	1.000
Hypodiploid	21.5	8.48	0.001
Amplification of 1q21	54.5	43.6	0.058
g70high	39.2	12.7	0.000
MRI1	74.9	77.3	0.612
7grp	60.5	23.6	0.000
Strata(train)	51.6	49.1	0.669

### MS Analysis Reveals the Potential Signaling Pathway for RFWD2 Function in MM

**FIGURE 2:**
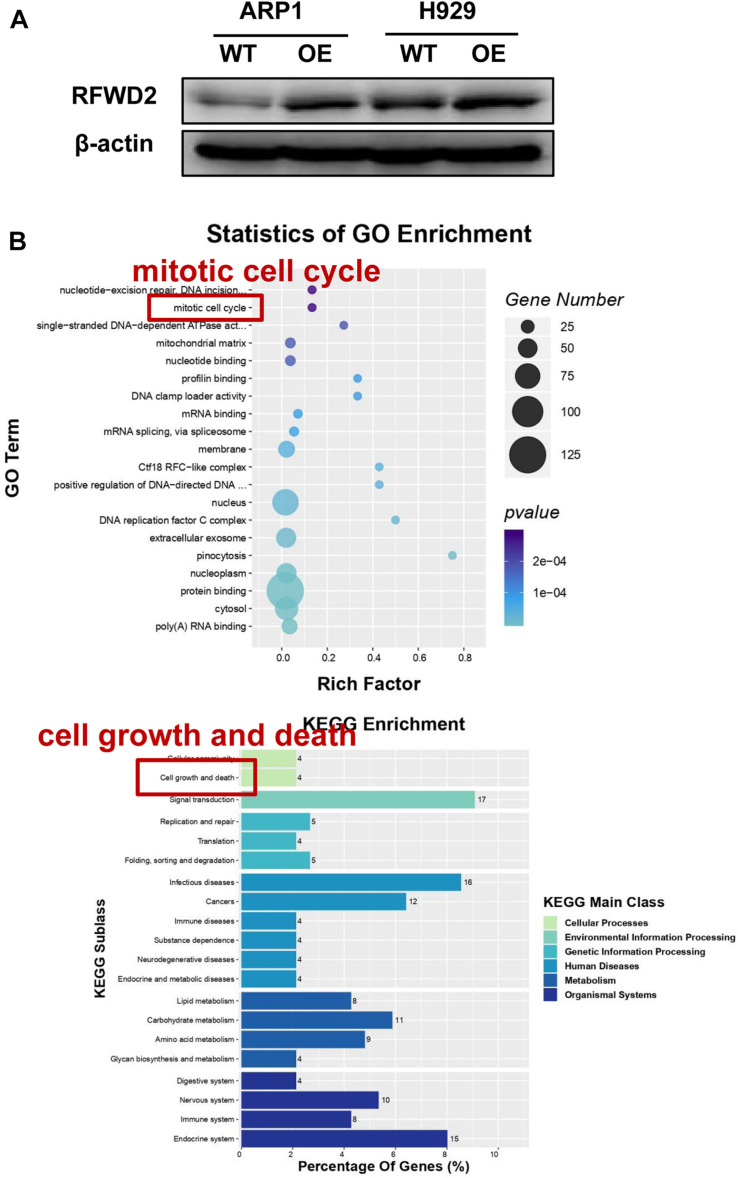
The potential signaling pathway of RFWD2 for MM biology is examined by MS. **(A)** WB analysis for validating RFWD2 expression in ARP1 and H929 cells transfected by RFWD2-cDNA (OE) vs wild type (WT) cells. **(B)** Gene ontology (GO) (up) and Kyoto Encyclopedia of Genes Genomes (KEGG) (down) pathway classification were performed in ARP1 WT/OE cell lines. The data were expressed as mean ± SD.

To address the potential role of RFWD2 in myeloma biology, we adopted two independent MM cell lines ARP1 and H929 as *in vitro* experimental models for MM. ARP1 and H929 cells were transfected with CRISPR lentiviral activation particles to functionally overexpress (OE) RFWD2. WB analysis confirmed the increment of RFWD2 expression in RFWD2 OE cells relative to wild-type cells (WT) serving as controls (Figure 2A). Furthermore, MS was conducted to assess activation of RFWD2-related signaling pathways. Representative gene ontology (GO) Biological Process terms and Kyoto Encyclopedia of Genes and Genomes (KEGG) pathways chosen from the most enriched charts were presented in Figure 2B, suggesting the top 20 most significantly enriched pathways. Above data indicated that the activation of two pathways related to RFWD2 in MM progression were mitotic cell cycle and cell growth and death, which would be basic guidance for further research on RFWD2.

### The Decrease of RFWD2 Hinders Cellular Proliferation in MM Cells

**FIGURE 3:**
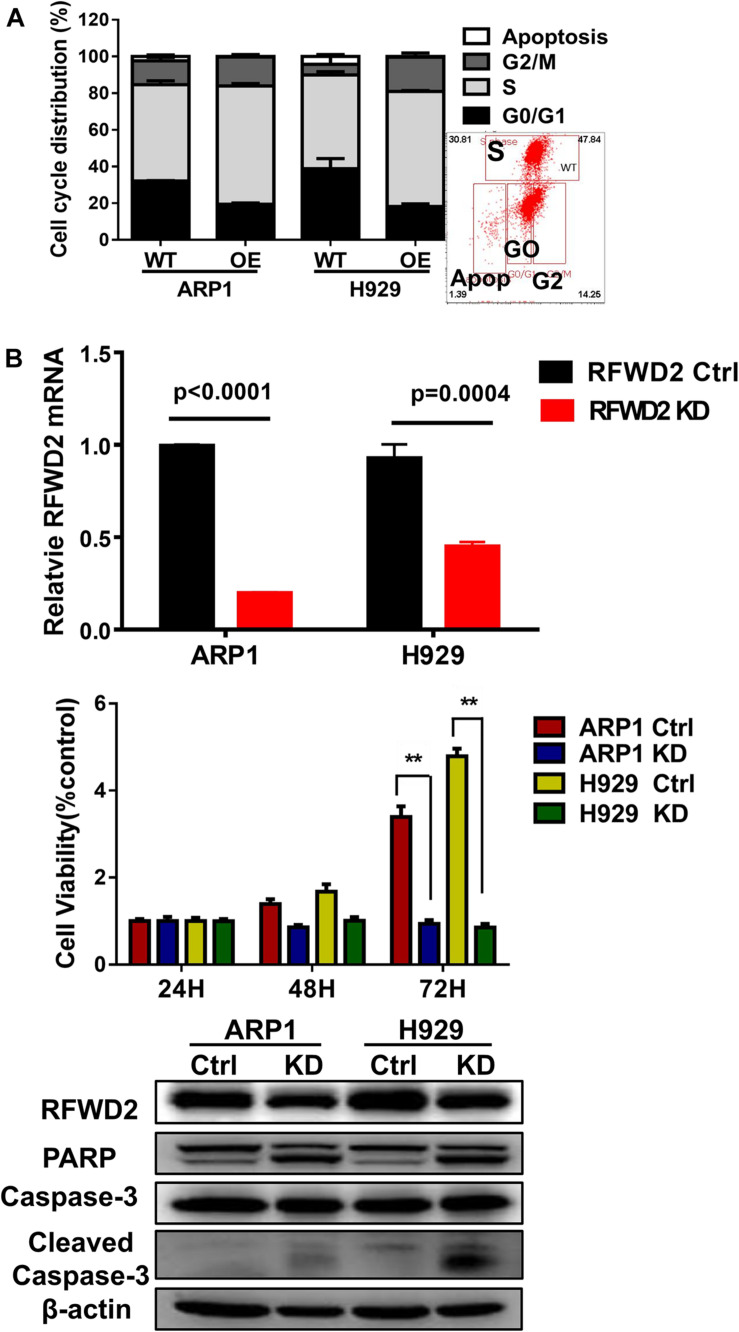
Inducible downregulation of RFWD2 suppresses MM cellular proliferation. **(A)** Cell cycle analysis of RFWD2-OE ARP1 and H929 MM cells compared to WT cells. **(B)** Up: Message levels of RFWD2 upon knocking down (KD) were assessed by using qRT-PCR method; Middle: MTT assay showed that silencing RFWD2 by shRNA remarkably inhibited MM cell growth rate; Down: WB analysis of RFWD2, PARP, Caspase-3 and cleaved Caspase-3 expression in ARP1 and H929 MM cells with or without RFWD2 KD. The data were expressed as mean ± SD, **P < 0.01.

In our previous paper, it has been illustrated that enforced expression of RFWD2 executed positive function in regulating MM cellular proliferation (Gu et al., 2020). Here, we continued to investigate the mechanism in depth. Flow cytometry analysis showed that the proportions of cells in the S phase were increased in RFWD2 OE cells relative to the controls (Figure 3A). Lentiviral shRNA transfection technology was conducted to knockdown the endogenous expression of RFWD2 in ARP1 and H929 cells. Then, qPCR and WB were recruited to validate the efficiency of shRNA, which demonstrated the significant decrease of RFWD2 at mRNA and protein levels in RFWD2-shRNA transfected MM cells (KD) compared to the WT cells (Figure 3B up and down). A prominent decrease of cell growth rate in ARP1 and H929 cells was provoked by silencing RFWD2 (*P* < 0.05) in a time-dependent manner (Figure 3B, middle), further confirming that RFWD2 facilitated MM cell proliferation. PARP and Caspase-3 have been authenticated as two key proapoptotic molecules in a broad spectrum of cancers. WB examination indicated that the expression of PARP and cleaved Caspase-3 expression was increased in RFWD2-shRNA cells compared to that in WT cells (Figure 3B down). Taken together, we further confirm that RFWD2 activation is critical for promoting MM cellular proliferation via controlling cell cycle and apoptosis *in vitro*.

### RFWD2 Mediates P27 Degradation to Influence MM Cell Growth

**FIGURE 4:**
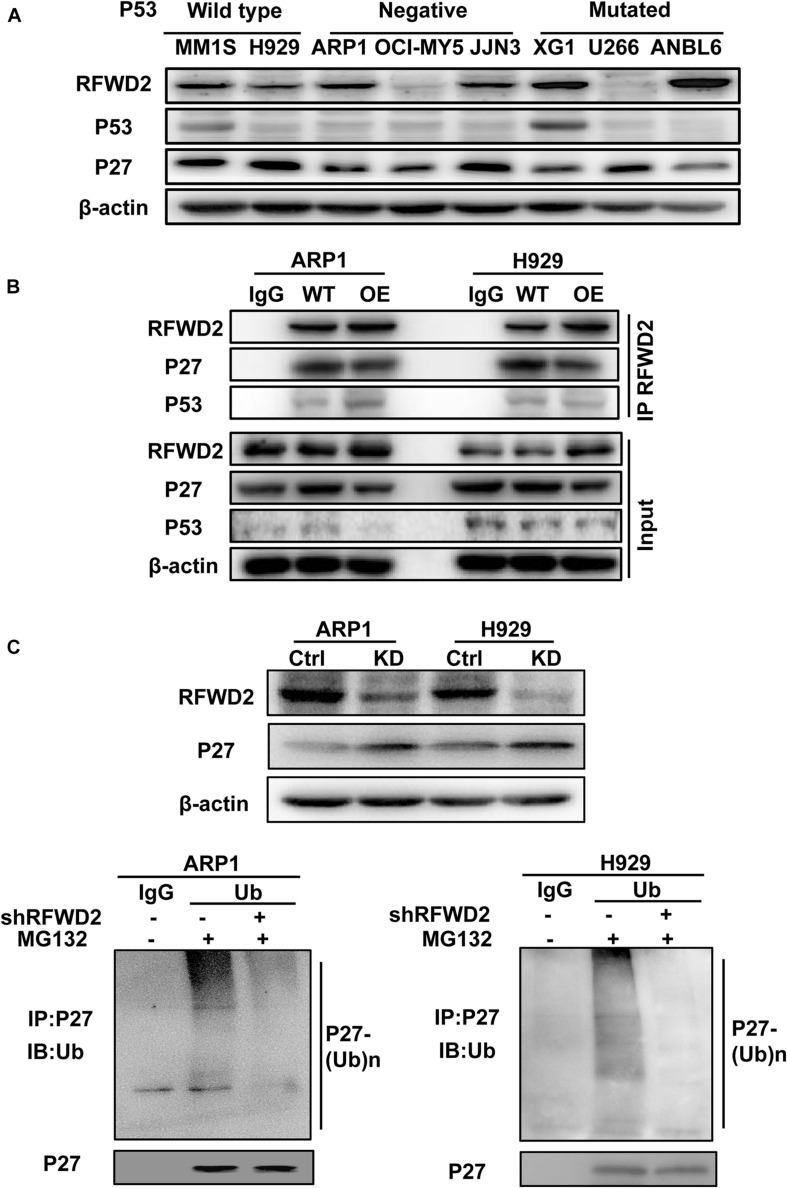
RFWD2 mediates P27 degradation to influence MM cell growth. **(A)** WB results showed the expression of RFWD2, P27 and P53 in 8 MM cell lines with wild-type, negative or mutated expression of P53. **(B)** Co-IP assay for the interaction between RFWD2 and P27, as well as P53 in RFWD2 WT/OE ARP1 and H929 cells. **(C)** Up: Detection of RFWD2 and P27 protein levels in WT/RFWD2-shRNA transfected MM cells; Down: Depletion of RFWD2 expression in MM cells resulting in reduction of ubiquitylated P27 expression.

Since RFWD2 is modulating both P27 and P53 (Dornan et al., 2004a; Ko et al., 2019), the two vital factors mediating cellular proliferation, we aim to identify which one is the major downstream factor of RFWD2. As Figure 4A shown, relatively higher level of P27 was ubiquitously observed in 8 MM cell lines with wild-type, negative or mutated expression of P53 (Xiong et al., 2008) by WB, while P53 expression was comparatively lower than P27 in 7 of 8 cells no matter mutated or not. More importantly, Co-IP assay demonstrated that the interaction between RFWD2 and P27 was more pronounced than with P53 (Figure 4B). Under overexpression of RFWD2, the interaction between P53 and RFWD2 did not increase and remained at a low level, supporting that P27 was the major target of RFWD2 in MM. The function of P27 is triggering cell cycle arrest by repressing cyclin-dependent kinase (CDK) activity (Sharma and Pledger, 2016; Fang et al., 2017), and P27 level is dominantly monitored by polyubiquitination, while RFWD2 acts as an E3 ubiquitin ligase. As proved by WB analysis, the protein level of P27 was up-regulated by blocking RFWD2 (Figure 4C up). After cells were treated with MG132, a reversible proteasome inhibitor, substantial increment of ubiquitylated P27 was shown by *in vitro* ubiquitylation assay. Additionally, the amount of ubiquitylated P27 in RFWD2 KD cells was well below that of the WT cells (Figure 4C down), implicating that RFWD2 participated in the ubiquitination modification and degradation of P27 through the proteasome pathway. On the basis of these observations, we propose that targeting RFWD2 impedes MM cellular proliferation via regulating the degradation of P27.

### RFWD2 Collaborates With RCHY1 E3 Ubiquitin Ligase to Meditate P27 Ubiquitination in MM

**FIGURE 5:**
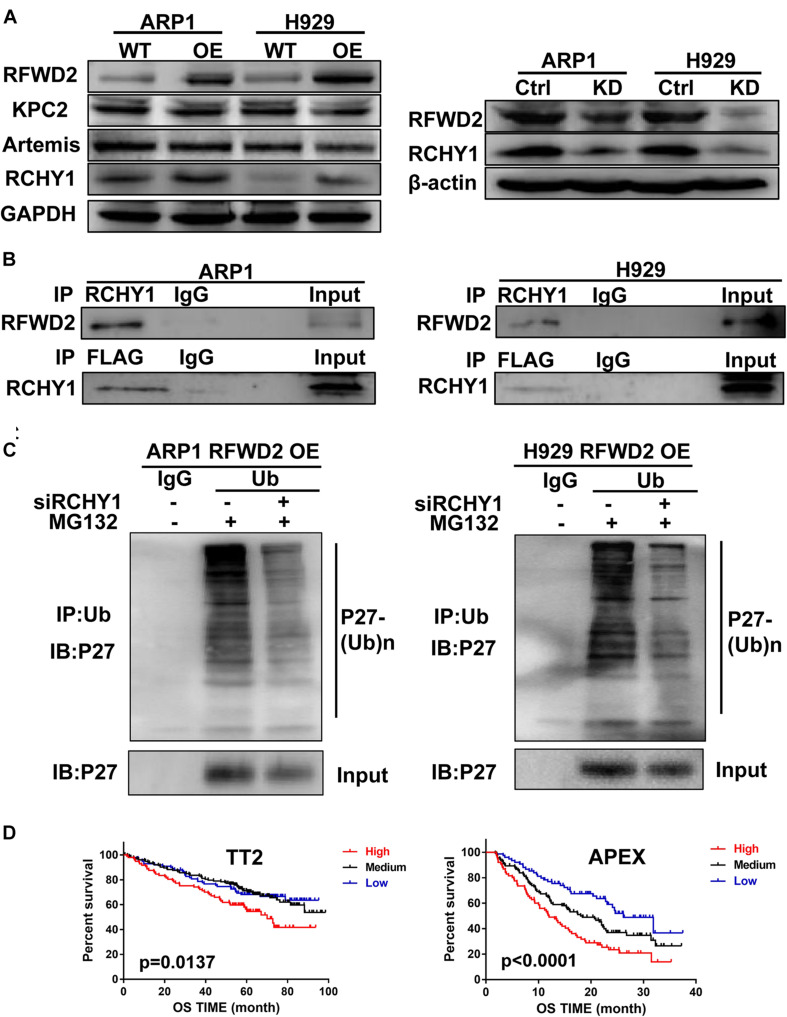
RFWD2 regulates P27 ubiquitination through interacting with RCHY1 E3 ubiquitin ligase in MM. **(A)** Left: Protein levels of E3 ubiquitin ligases (KPC2, Artemis and RCHY1) of P27 were measured by WB in RFWD2 WT/OE ARP1 and H929 cell lines; Right: Protein levels of RCHY1 E3 ubiquitin ligase were detected in ARP1 and H929 cells with or without RFWD2 KD. **(B)** The physical interaction between RFWD2 and RCHY1 was identified by Co-IP experiment. **(C)** The ubiquitination level of P27 was detected in RFWD2 OE cells upon transfection of RCHY1 siRNA or not. **(D)** Kaplan-Meier analysis for MM patients with different levels of RFWD2 and RCHY1 expression. The patient survival classified by high/low RFWD2 expression and high/low RCHY1 expression were described. The cases were designated as high expressers while both RFWD2 and RCHY1 message were above (indicated in red) the medium level, or stratified as low expressers while both RFWD2 and RCHY1 message were below (blue) the medium level in the TT2 and APEX dataset. All remaining cases (RFWD2^*High*^/RCHY1^*Low*^ or RFWD2^*Low*^/RCHY1^*High*^) were stratified as medium expressers (black).

Kip ubiquitination-promoting complex (KPC) complex, RING-finger and CHY-zinc-finger domain-containing protein 1 (RCHY1, also known as Pirh2) and CRL4DDB2-Artemis E3 ligases are identified as E3 ubiquitin ligases of P27 (Zhao et al., 2013; Masumoto and Kitagawa, 2016; Dobashi et al., 2017; Li et al., 2019). To find the detailed factor by which RFWD2 mediated P27 degradation, we examined the correlation between RFWD2 and the three E3 ubiquitin ligases. WB analysis showed only RCHY1 expression was increased in RFWD2 OE cells (Figure 5A left), and the expression of RCHY1 was reduced in RFDW2 KD cells (Figure 5A right). Then, the physical interaction between RFWD2 and RCHY1 was verified by Co-IP assay. With using FLAG antibody for IP and RCHY1 antibody for IB, RCHY1 band could be detected and vice versa (Figure 5B). Strikingly, intervention of RCHY1 by siRNA resulted in decreased ubiquitination of P27 in RFWD2 OE cell lines (Figure 5C) that validated RFWD2 mediating P27 expression through interacting with RCHY1 E3 ubiquitin ligase. In addition, Figure 5D presented that patients in TT2 or APEX cohorts with a high/high co-expression of RFWD2-RCHY1 experienced poor survival outcomes relative to patients with low/low co-expression or medium expression. The findings indicate a potentially synergistic effect of RFWD2 and RCHY1 on MM patient prognosis.

### Reduction of RFWD2 Reverses BTZ Resistance in MM Xenograft Model

**FIGURE 6:**
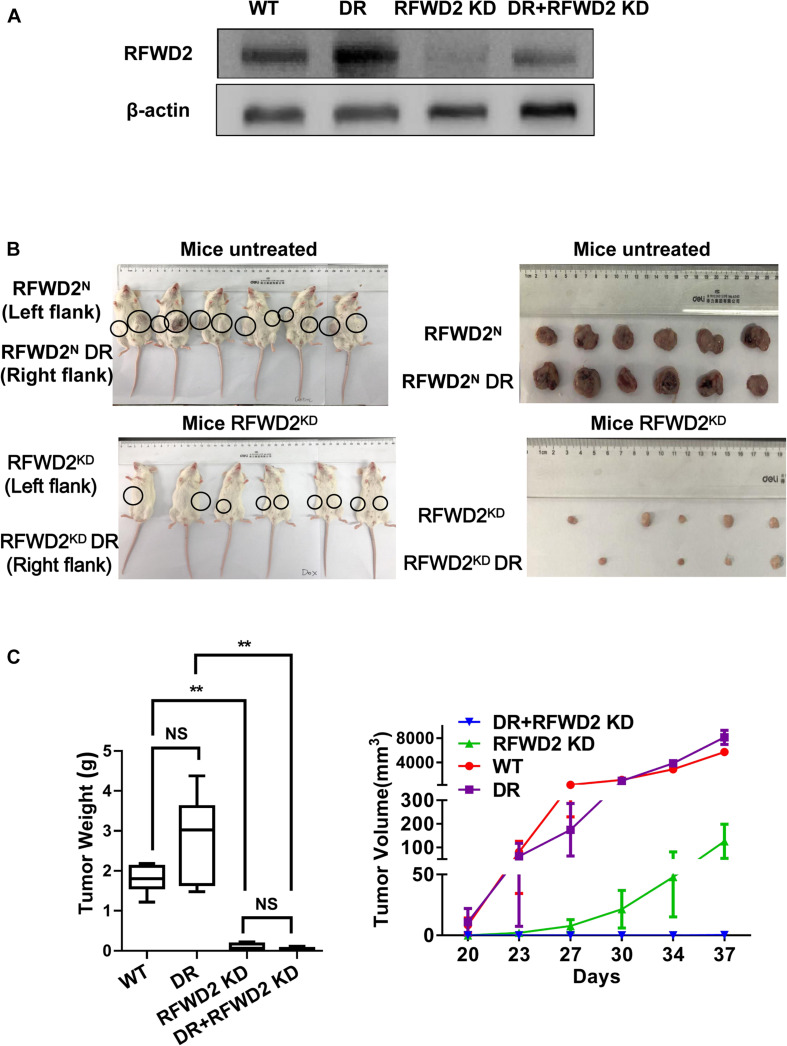
Reduction of RFWD2 reverses BTZ resistance in MM xenograft model. **(A)** WB assay was conducted to evaluate RFWD2 protein levels in 8226 WT, 8226 RFWD2 KD, 8226/BTZ and 8226/BTZ RFWD2 KD xenografts. **(B)** Photographic images of xenograft-bearing mice (left) and tumor growth (right) from each group were captured. **(C)** Left: Mean tumor weight in the four experimental groups at day 28 post implantation of the specified MM cells; Right: Time course of tumor growth in myeloma xenografts received 8226 WT, 8226 RFWD2 KD, 8226/BTZ and 8226/BTZ RFWD2 KD cells in each flank. The data were expressed as mean ± SD, **P < 0.01, NS, no significance.

Our previous research has shed light on the vital role of RFWD2 in MM PIs resistance; we further verified whether RFWD2 inhibition could overcome drug resistance *in vivo* (Gu et al., 2020). To this end, RFWD2 shRNA was transfected to 8226 WT and 8226 BTZ-resistant (DR) cells. DOX was applied to induce shRNA expression. The 8226 WT and 8226 DR cells with genetic ablation of RFWD2 were injected into NOD-SCID mice with or without DOX stimulation. Elevated amounts of RFWD2 protein were observed in the DR group compared with the untreated WT group, while RFWD2 expression was downregulated in both WT and DR groups by shRNA (Figure 6A). RFWD2 KD tumors in both WT and DR groups harvested at study endpoint were extremely smaller than the tumors with normal expression of RFWD2 (Figure 6B). The similar trend was also exhibited in tumor weight (Figure 6C left) and volume (Figure 6C right), suggesting that RFWD2 inhibition could decrease the tolerance to BTZ *in vivo*. Combined with the data *in vitro*, we conclude that targeting RFWD2 offers a suitable therapeutic approach for halting MM progression and overcoming drug resistance.

## Discussion

The ubiquitin-proteasome system (UPS) plays a key role in regulating the levels and activities of a multitude of proteins as well as modulation of cell cycle, gene expression, cell survival, cell proliferation and apoptosis in MM (Crawford et al., 2020). MM cells typically produce a substantial amount of paraprotein and deeply rely on the UPS to maintain cellular homeostasis (Franqui-Machin et al., 2018). Ubiquitination is a process in which ubiquitin molecules bind to the target protein under the action of E1 ubiquitin activating enzyme, E2 ubiquitin conjugating enzyme and E3 ubiquitin ligase to modify the ubiquitination of the target protein (Mulder et al., 2016). Preclinical studies have highlighted a rich source of E3 ubiquitin ligases rendering resistance to PIs in MM cells and developed anti-E3s based cancer therapeutics for MM treatment (Zhang et al., 2016; Chen et al., 2018; Barrio et al., 2020; Huang et al., 2020). In the current study, we introduced an E3 ubiquitin ligase RFWD2 located at the long arm of chromosomal position 1q25, which is of particular interest in MM (Shaughnessy, 2005; De Boussac et al., 2020). The data of gene expression profiling from 3 independent MM cohorts (TT3, HOVON65 and APEX) were analyzed, which indicated that high RFWD2-expression patients were intimately associated with adverse prognosis, disease relapse and myeloma cell proliferation, as consistent with our previous results in TT2 and GMMG-HD4 cohort (Gu et al., 2020). All these provide ample experimental evidence for RFWD2 acting as an attractively molecular predictor in advanced myeloma.

Since RFWD2 governs a series of biological activities, we further develop a deeper knowledge surrounding RFWD2 and MM using lentivirus knockdown and overexpressing approaches. MS analysis showed that the impact of RFWD2 on cell cycle, cell growth and death were involved in MM process. Inducible downregulation of RFWD2 elicited an apparent decrease in growth rates of ARP1 and H929 cells via regulating cell cycle and apoptosis, which made a complementary to our previous report on overexpression of RFWD2 (Gu et al., 2020). The function of RFWD2 differs in diverse tumors largely depending on degradation of its specific downstream substrates, such as c-Jun (Migliorini et al., 2011), FOXO1 (Kato et al., 2008), P53 (Dornan et al., 2004b) and ETS transcription factors (Vitari et al., 2011). Several research have highlighted the significance of P53 and P27 for cell cycle and apoptosis involved in RFWD2-driven carcinogenesis (Choi et al., 2015a; Ka et al., 2018). Guided by the data of WB and Co-IP assessment on P53 and P27, we found the higher expression of P27 and the stronger linkage of RFWD2 and P27, which suggested that P27 was the major target of RFWD2 in MM. Next, we unraveled that depletion of RFWD2 impaired ubiquitination and degradation of P27 to induce cell cycle arrest, thereby blunt MM cell growth.

To query the mechanism underlying RFWD2-induced tumorigenesis via mediating P27, we further evaluated the moderator involved in the interaction between RFWD2 and P27. Mounting evidence has pointed out that P27 is predominately regulated by KPC2 at G1 phase, leading to translocation-coupled cytoplasmic ubiquitination (Kamura et al., 2004; Masumoto and Kitagawa, 2016). In addition, P27 is found to be degraded through CRL4DDB2-Artemis E3 ligases (Zhao et al., 2013). Recently, RCHY1 has been proved to act as a novel E3 ubiquitin ligase for P27 via directly binding and ubiquitylating P27 from late G1 to S phase (Shimada et al., 2009; Masumoto and Kitagawa, 2016). Both RCHY1 and RFWD2 are RING type E3 ubiquitin ligases. RFWD2 serves as one of the RCHY1-binding partners, and functional interplay between them can inhibit P53 activity synergistically in non-small cell lung cancer (Wang et al., 2011). We first explored these specific E3 ubiquitin ligases in MM and found that the positive relationship was observed only between RCHY1 and RFWD2. Co-IP assay was employed to further validate the physical interaction of RCHY1 and RFWD2. In addition, silencing RCHY1 by siRNA abolished the ubiquitination of P27 in RFWD2 OE cell lines. However, the study performed in human 293T, HeLa and MDA-MB231 cells demonstrated that the E3 ubiquitin ligases of P27 like RCHY1 did not participate in RFWD2-mediated P27 degradation (Choi et al., 2015b). The reason of the two distinctive conclusions may be attributed to diverse genetic backgrounds, molecular manipulators and signal pathways presented in different types of cancer. Notably, we found that the increased co-expression of RFWD2 and RCHY1 yielded a severe detrimental impact on the prognosis of MM patients. Collectively, we infer that RFWD2 mediates P27 ubiquitination to facilitate MM progression by interacting with the RCHY1 E3 ubiquitin ligase, which contributes to a potentially novel mechanism regarding RFWD2-driven carcinogenesis.

It has been well recognized that P27 is one of the major targets of PIs like BTZ (Iskandarani et al., 2016; Fotouhi et al., 2019), while RFWD2 is the key regulator of P27. We have proved that targeting RFWD2 potentially overcomes BTZ resistance *in vitro.* To put forward our findings into *in vivo* study, we adopted paired 8226 WT and BTZ-resistant cells with RFWD2 KD in MM xenograft model. Both 8226 WT or 8226/BTZ RFWD2 KD tumor expansion were outstandingly lagged behind their corresponding partner control, indicating that targeting RFWD2 could repress tumor expansion and overcome BTZ resistance both *in vitro* and *in vivo*.

In summary, we provide more preclinical evidence to strengthen the notion that targeting RFWD2 can inhibit MM cellular proliferation and drug resistance to proteasome inhibitor via regulating P27. In addition, our findings provide important insights into the mechanism by which RFWD2 and RCHY1 collaborate to negatively regulate P27 stability, indicating that blocking the RFWD2-RCHY1 signaling axis is a feasible strategy with reduced P27 to potentiate PIs therapy for combating MM. The development of advanced techniques on screening chemical inhibition of RFWD2 for MM therapy is entered into new research frontier.

## Data Availability Statement

The original contributions presented in the study are publicly available. This data can be found here: the ProteomeXchange Consortium: PXD024507.

## Ethics Statement

The animal study was reviewed and approved by Institutional Ethics Review Boards of Nanjing University of Chinese Medicine.

## Author Contributions

CG and MG conceived the manuscript and provided critical input. MG drafted the manuscript. PD, SY, MG, LF, and YZ performed the experiments. ZZ provided technical counseling on experiments. CG and YY reviewed the data and edited the manuscript. All authors read and approved the final manuscript.

## Conflict of Interest

The authors declare that the research was conducted in the absence of any commercial or financial relationships that could be construed as a potential conflict of interest.
